# A novel indole-based conjugated microporous polymer for highly effective removal of heavy metals from aqueous solution *via* double cation–π interactions†

**DOI:** 10.1039/c9ra07970j

**Published:** 2019-12-06

**Authors:** Qiang Wang, Rui Li, Xiao Ouyang, Guojun Wang

**Affiliations:** Key Laboratory of Superlight Materials and Surface Technology, Ministry of Education, College of Materials Science and Chemical Engineering, Harbin Engineering University Harbin 150001 China wang5347@hrbeu.edu.cn

## Abstract

A novel indole-based conjugated microporous polymer (PTIA) with three coplanar indole units, designed and synthesized by an oxidative coupling reaction, was utilized as a platform for removing heavy metals. Owing to the conjugation of the three coplanar indoles, the highly electron-rich large π planes can simultaneously attract six heavy metal atoms *via* double cation–π interactions, endowing this microporous material with remarkable heavy metal adsorption capacity and efficiency.

## Abstract 2

A novel indole-based conjugated microporous polymer (PTIA) with three coplanar indole units, designed and synthesized by an oxidative coupling reaction, was utilized as a platform for removing heavy metals.

## Graphical abstract

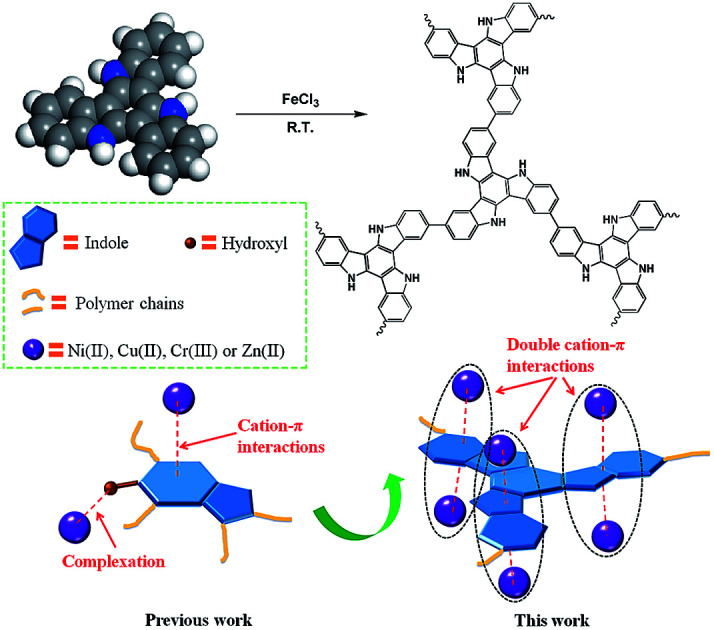

## Introduction

In recent years, the health of human beings has been endangered by heavy metal ions, and it is therefore very important to remove heavy metals from aqueous solutions due to their toxicity and bioaccumulation.^1–4^ Moreover, excessive intake of heavy metal ions, for example, Ni(ii),^5^ Cu(ii),^6^ Zn(ii)^7^ and Cr(iii)^8^ is harmful to humans and other species. A wide range of technologies have been investigated for heavy metal ion removal from water including chemical precipitation,^9^ organic chelation,^10,11^ biological removal,^12^ ion-exchange,^13,14^ membrane separation^15^ and adsorption.^16,17^ Among these approaches, adsorption is found to be the most attractive due to its advantages of relative simplicity, easy scale-up and high efficiency over a wide concentration range.^18,19^ Developing novel efficient, economical, green and environment-friendly adsorbents for heavy metal ions has been considered as an effective way to mitigate the deterioration of the environment.^20–23^ Conjugated microporous polymers (CMPs) have many specific features such as large surface area, high porosity, adjustable pore size, thermal and chemical stability, showing great advantage in removal of heavy metals.^24–26^ However, the inherent physisorptive metal-adsorption mechanism makes them inevitably suffer from low adsorption capacity and efficiency hence severely hampers their practical applications. Compared with adjusting the pore parameters and surface area, according to the recent research results, the incorporation of some specific functional groups or heteroatoms into the microporous framework to improve the heavy metals binding affinity has been revealed to be a simple and effective method to enhance the heavy metals adsorption capacity and efficiency.^27,28^ As a typical aromatic compound, indole possesses more rich electronic structure than general aromatic structure,^29–33^ which makes it more easily to form cation–π interaction with cations. Chang *et al.* has found that the 4-hydroxyindole-formaldehyde aerogel (4-HIFA) containing hydroxyl and electron-rich indole ring possessed strong affinity for heavy metals *via* the synergistic effects of complexation and cation–π interactions,^34^ however, the single cation–π interaction means that one indole plane can only attract one heavy metal and the additional functional groups would consume the occupied volume of the porous frameworks, leading to less improvement in the heavy metal adsorption capacities for these indole-based porous materials with multi-functional groups. Based on the work of Chang *et al.*,^34^ therefore, to better utilize the cation–π interactions for developing new heavy metals adsorbent with both excellent adsorption capacity and efficiency still remains attractive prospects and great challenge.

**Scheme 1:**
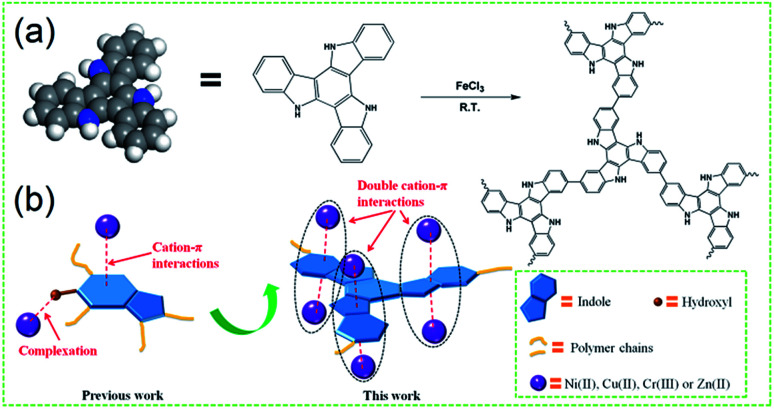
(a) The coplanar structure of the repeating unit and the synthetic route of the indole-based conjugated microporous polymer (PTIA), (b) schematic illustration of heavy metals adsorption using PTIA *via* double cation–π interactions.

Herein, a new type of indole-based conjugated microporous polymer (PTIA) was designed and synthesized in the presence of the FeCl_3_ catalyst by a facile direct oxidative coupling reaction (Scheme 1a). We expected that the conjugation of coplanar three indole would form a highly electron-rich large π plane which can simultaneously attract six heavy metal *via* the double cation–π interactions, resulting in a great increase heavy metal adsorption capacity and efficiency (Scheme 1b). Because the texture properties of the CMPs are mainly governed by the design or selection of a specific monomer, herein, we used the indole-based monomer 10,15-dihydro-5*H*-diindolo[3,2-*a*:3′,2′-*c*]carbazole (TAT) (Fig. S1, ESI†), which has a coplanar structure. The TAT utilized in the polymerization was synthesized in a single step using 2-indolinone as the monomers. The synthesized TAT was polymerized in the presence of the FeCl_3_ catalyst by direct oxidative coupling reaction, and the material preparation and characterization are detailed in the ESI†.

## Experimental

The main materials, measurements, synthetic routes of the TAT and PTIA, pH effect on the removal of Ni^2+^, Cu^2+^, Cr^3+^ and Zn^2+^ by PTIA, kinetics data and sorption data of PTIA toward Ni^2+^, Cu^2+^, Cr^3+^ and Zn^2+^, adsorption cycle test and simulation method are described in ESI.†

## Results and discussion

As shown in Fig. S2 and S3,† the atoms of TIA belonged to aromatic H and C. The resulting PTIA was characterized by Fourier transform infrared and ^13^C CP/MAS NMR, and the results were in good agreement with the proposed structures (Fig. S4 and S5, ESI†). The FTIR spectrum of the PTIA is shown in Fig. S4,† in which the absorption peaks at about 3423 cm^−1^ correspond to the structure of NH groups. The peak at 1606 cm^−1^ is attributed to the skeleton vibrations of conjugate structures in the network. As shown in Fig. S5,† the broad peaks at 150–100 ppm are ascribed to the indole group carbons.

**Fig. 1:**
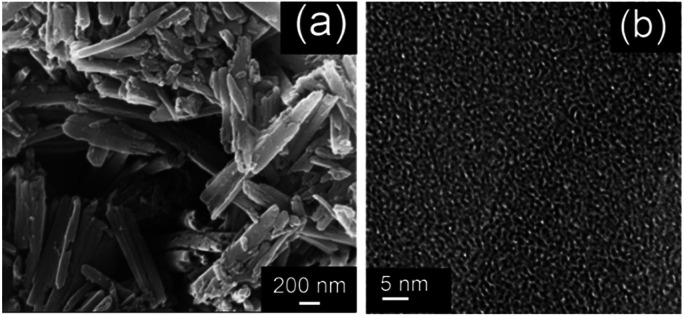
Micro-structures of the PTIA network. (a) SEM, (b) TEM.

The SEM of the PTIA displays that the material consists of aggregated particles with sub-micrometer sizes (Fig. 1a), and the TEM image (Fig. 1b and S7†) is indicative of porous structures of the PTIA material that are essential requirements for removal of heavy metals. The nitrogen adsorption–desorption isotherms of PTIA measured at 77 K is shown in Fig. S6,† displaying a typical curve of type I,^35^ which is consistent with their expected microporous nature. There are hysteresis loops at relative pressures of 0.2–0.95, suggesting the presence of mesopores and macropores in the sample,^36^ in good agreement with those observed with the SEM technique (Fig. 1a). The calculation for data showed that the Brunauer–Emmett–Teller (BET) specific surface area of the PTIA is up to 139 m^2^ g^−1^. Additionally, nonlocal density functional theory (NLDFT) was used to approximate the pore size distributions (PSD) of PTIA employing the cylindrical pore-oxide surface model.^37^ The calculation yielded an average pores size of ∼1.9 nm and a sharp peak ∼1.92 nm.

It was expected that the resulting PTIA with the conjugation of coplanar three indole would form a highly electron-rich large π plane which can simultaneously attract six heavy metal *via* the double cation–π interactions, which inspires us to investigate its heavy metal ions adsorption capacity and efficiency.

The pH of solution is one of the most important parameters affecting sorption process. Fig. S8† shows the dependences of the adsorption capacity for Ni^2+^, Cu^2+^, Cr^3+^ and Zn^2+^ on the pH value (2.0–6.0) over PTIA in order to eliminate the effect of precipitation at higher pH values. Notably, that the ions adsorption of Ni^2+^, Cu^2+^, Cr^3+^ and Zn^2+^ was increased with the increase of pH value from 2.0 to 6.0. The phenomenon could be explained that at lower pH, the relatively high H^+^ concentration would strongly compete with metal ions for the adsorption sites, resulting in low adsorption capacity. With the increase of pH, the competition between H^+^ and other cations could be neglected. In view of the fact that the precipitation of Ni^2+^, Cu^2+^, Cr^3+^ and Zn^2+^ takes place at pH ≥ 7, pH 6.0 was selected as the optimum pH for the following batch experiments to eliminate the effect of precipitation.

**Fig. 2:**
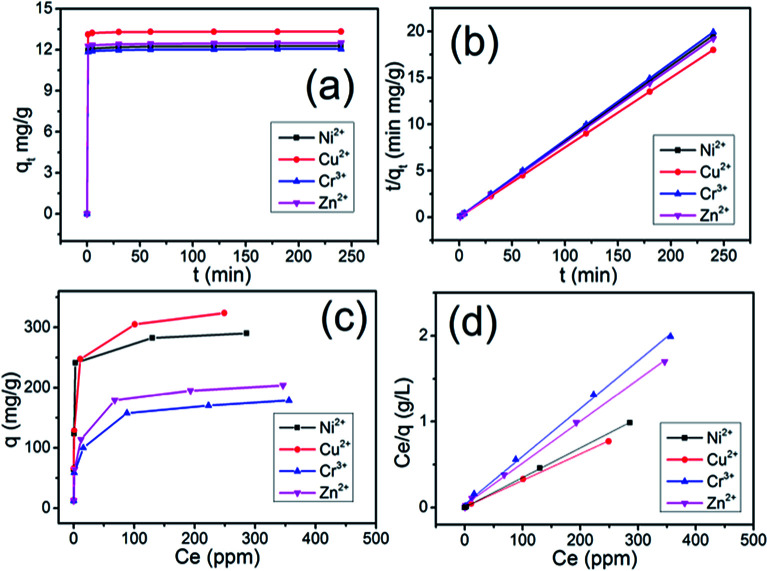
Adsorption kinetics curves and sorption isotherms for M^*n*+^ (M^*n*+^ = Ni^2+^, Cu^2+^, Cr^3+^, Zn^2+^) by PTIA: (a) sorption capacity (*q*_*t*_) with time, (b) pseudo-second-order kinetic plots for sorption, Langmuir equilibrium isotherms were derived from equilibrium concentration (*C*_e_, ppm), plotted against the adsorption capacity (c) *q* (mg g^−1^) and (d) *C*_e_/*q*_e_ (g L^−1^).

The adsorption kinetics of the Ni^2+^, Cu^2+^, Cr^3+^ and Zn^2+^ ions by PTIA was investigated in order to study adsorption rate and pathways of adsorption until equilibrium was reached (240 min after starting adsorption). The results (Tables S1–S4, ESI†) and sorption kinetics curves (Fig. 2a) show rapid uptake rates and high removal efficiency. Within 1 min, the PTIA achieved ≥97% removal rates and *K*_d_ values of >10^4^ mL g^−1^ for Ni^2+^ (Table S1†) and Cu^2+^ (Table S2†). Within 5 min, the PTIA achieved ≥98% removal rates and *K*_d_ values of >10^4^ mL g^−1^ for Ni^2+^ (Table S1†) and >10^5^ mL g^−1^ for Cu^2+^ (Table S2†). Within 30 min, the PTIA achieved ≥99% removal rates and *K*_d_ values of >10^5^ mL g^−1^ for Ni^2+^ (Table S1†) and for Cu^2+^ (Table S2†). For the Cr^3+^ and Zn^2+^ ions (Tables S3 and S4†), the adsorption is slightly slow but still has a 97.65% and 98.29% removal rate in 30 min, respectively. The adsorptions for all of the four ions reach equilibrium within ∼1 min (Fig. 2a), which is more faster than that of the previously reported indole-based aerogel.^34^ The PTIA shows faster heavy metal ions adsorption due to the conjugation of coplanar three indole would form a relatively large binding area to capture heavy metal ions.

The removal rate can be determined in two different ways: pseudo-first-order and pseudo-second-order mechanisms, which were defined as follows:^38^

Pseudo-first-order:

1 ln(*q*_e_ − *q*_*t*_) = ln *q*_e_ − *k*_1_*t*

Pseudo-second-order:2
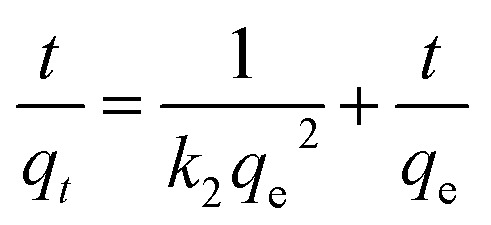
where *q*_e_ (mg g^−1^) is the adsorbed amount per unit mass of adsorbent at equilibrium and *q*_*t*_ (mg g^−1^) is the adsorbed mass at time *t*, while *k*_1_ (min^−1^) and *k*_2_ (g mg^−1^ min^−1^) are corresponding equilibrium rate constants. The *k*_1_ value was obtained by plotting ln(*q*_e_ − *q*_*t*_) against *t* and the *k*_2_ by plotting *t*/*q*_*t*_ against *t*. The linear relationship of *t*/*q*_*t*_*versus t* was presented in Fig. 2b. From the kinetic parameters of Ni^2+^, Cu^2+^, Cr^3+^ and Zn^2+^ (Table S5†), the calculated sorption capacities (*q*_e,cal_) derived from the pseudo-second-order model are quite close to corresponding experimental values (*q*_e,exp_). The fit coefficient (*R*_2_) of 1 indicates the adsorption is well fitted with the pseudo-second-order kinetic model, suggesting a chemisorption process.^39^

Uptake capacity toward Ni^2+^, Cu^2+^, Cr^3+^ and Zn^2+^ by PTIA from aqueous solutions was studied with the batch method at room temperature. The maximum adsorption capacity of the material was determined from an adsorption equilibrium study. The Ni^2+^ capture by PTIA was found to increase successively with increasing concentration (10–500 ppm, Table S6†). Over a wide range of the initial concentration (10–100 ppm), the Ni^2+^ removal rates reached values of >99%, with the *K*^Ni^_d_ values ranging from 2.2 × 10^5^ to 1.4 × 10^7^ mL g^−1^. The maximum removal capacity (*q*_m_) for Ni^2+^ reached ∼290.0 mg g^−1^. This is an exceptionally high capacity competing with those of the best absorbers such as PMCNa hybrid hydrogels (224 mg g^−1^),^40^ for which adsorption capacities of various adsorbents are shown in Table S11.† We also checked the adsorption of the PTIA material for Cu^2+^ in the range of concentrations 10 ppm to 500 ppm (Table S7†), and found that there are >99% removal rates in the initial concentration (10–100 ppm), and ∼323 mg g^−1^ maximum adsorption capacity, this value is still very high compared to reported adsorbents (Table S11†). For Cr^3+^ and Zn^2+^, the maximum adsorption capacity is found to be relatively lower at ∼178.8 mg g^−1^ and 203.8 mg g^−1^ (Tables S8 and S9†), however those value is still very high compared to reported adsorbents (Table S11†), which is higher than that of the previously reported indole-based aerogel.^34^ The PTIA shows higher heavy metal ions adsorption due to the conjugation of coplanar three indole would form a highly electron-rich large π plane which can simultaneously attract six heavy metal *via* the double cation–π interactions.

A Langmuir isotherm is used to describe the experimental data of Ni^2+^, Cu^2+^, Cr^3+^ and Zn^2+^. In this model, the adsorbate moieties (Ni^2+^, Cu^2+^, Cr^3+^, Zn^2+^) are assumed to undergo monolayer type coverage of the sorbent on an adsorbent surface. Once an adsorption site is occupied, no further adsorption can happen at the same site. The Langmuir isotherm model is listed as equation:3
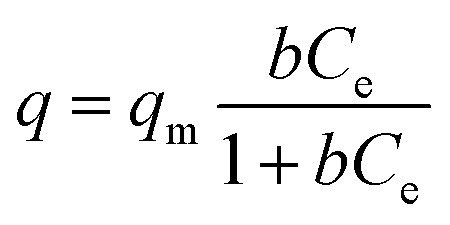
where *q* (mg g^−1^) is the equilibrium adsorption capacity of Ni^2+^, Cu^2+^, Cr^3+^ and Zn^2+^ adsorbed, *C*_e_ (mg L^−1^) is the Ni^2+^, Cu^2+^, Cr^3+^ and Zn^2+^ concentration at equilibrium, *q*_m_ (mg g^−1^) is the theoretical maximum sorption capacity. The equilibrium adsorption isotherms are shown in Fig. 2c, with the Ni^2+^, Cu^2+^, Cr^3+^ and Zn^2+^ equilibrium concentration ranging from 0.0005 to 356 ppm. The experimental data of uptake capacity for Ni^2+^, Cu^2+^, Cr^3+^ and Zn^2+^ are fitted well with the Langmuir isotherm model of eqn (3), see Fig. 2d. According to the Langmuir isotherm model, the expected capacity *q*_m_ of 289.9.5 mg g^−1^ for Ni^2+^, of 323.6 mg g^−1^ for Cu^2+^, of 179.9 mg g^−1^ for Cr^3+^ and of 205.3 mg g^−1^ for Zn^2+^, which are consistent with the experimental value of 290.0 mg g^−1^ for Ni^2+^, of 323.8 mg g^−1^ for Cu^2+^, of 178.8 mg g^−1^ for Cr^3+^ and of 203.8 mg g^−1^ for Zn^2+^. The large correlation coefficient (*R*^2^ > 0.99) shows a good fit with the Langmuir isotherm, suggesting a monolayer adsorption^41^ on the PTIA (Table S10†).

Fig. S9† shows Ni^2+^, Cu^2+^, Cr^3+^ and Zn^2+^ adsorption recycled for 4 times, after 1 cycles reused, metal ions adsorption decreased most, which may due to that part metal ions, that not be desorbed by HCl, occupied part of the adsorption sites. The metal ions adsorption for regeneration 2, 3 and 4 times were relatively close to each other. After 4 cycles reused, the amount of metal ions adsorption for Ni^2+^, Cu^2+^, Cr^3+^ and Zn^2+^ ions was 90, 103, 75 and 92 mg g^−1^, which was 74.6%, 80.2%, 75.0% and 81.4% of adsorption for the fresh sample, respectively. The excellent recyclability of the metal ions on PTIA is very helpful for practical applications, suggesting the long-term use in water purification.

**Fig. 3:**
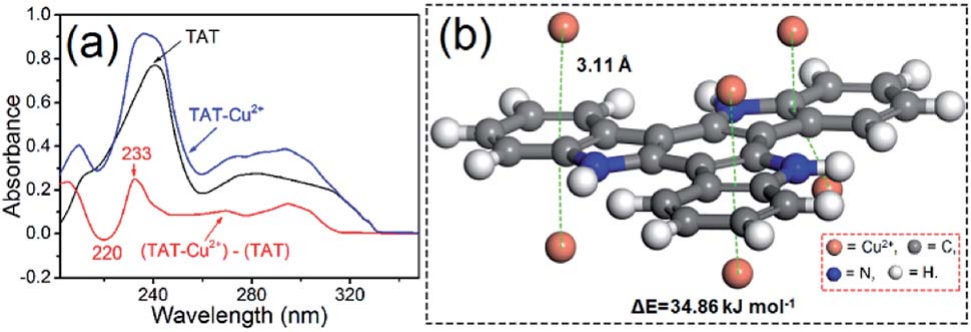
(a) UV absorption spectra of TAT (100 μM) and its 1 : 1 complex with Cu^2+^ at pH 7 (1 mM HEPES buffer), (b) fully optimized geometries of model interactions calculated using DFT method: the double cation–π conformation of PTIA with Cu^2+^.

To identify the interaction between PTIA samples and heavy metal ions during the adsorption process, UV absorption was performed. Fig. 3a shows the absorption spectra of 10,15-dihydro-5*H*-diindolo[3,2-*a*:3′,2′-*c*]carbazole (TAT) in the absence and presence of equimolar Cu^2+^, together with the difference spectrum TAT–Cu^2+^ minus TAT. The difference spectrum TAT–Cu^2+^ (red line) reveals a negative band at 220 nm and a positive band at 233 nm attributable to a Cu^2+^–TAT interaction. An analogous UV difference spectrum with a negative/positive band pair around 220/230 nm has been observed for an indolyl model compound of the cation–π interaction,^42^ a cation–π interaction between a positively charged His imidazole ring and a nearby Trp indole ring has also been reported to produce an analogous band pair.^43^ The similarity of the UV difference spectrum of TAT–Cu^2+^ with those reported previously for other types of cation–π interactions indicates that the TAT interaction in TAT–Cu^2+^ is also categorized as a cation–π interaction. To elucidate the adsorption mechanism, the Density Functional Density (DFT) calculations were performed to investigate the interactions between PTIA and Cu^2+^,^44–46^ the calculations were detailed in ESI.† The snapshot of Cu^2+^ adsorption are shown in Fig. 3b. The figure shows a minimized geometry of the model compound. The equilibrium conformation of Cu^2+^–TAT is six electron-deficient Cu^2+^ and the coplanar three indole ring forming sandwichy cation–π configuration with the distance of 3.11 Å, and the computational binding energy is 34.86 kJ mol^−1^ (Fig. 3b). Cu^2+^ can be easily and rapidly adsorbed on the coplanar three indole ring due to its relatively large binding area through double cation–π interactions.

## Conclusions

In summary, we have rationally designed a new type of indole-based conjugated microporous polymer (PTIA) that is easily prepared in the presence of the FeCl_3_ catalyst by a facile direct oxidative coupling reaction. We have demonstrated that the conjugated microporous polymer could be used as a high effective and extraction material for heavy metals from aqueous solution through the conjugation of coplanar three indole would form a highly electron-rich large π plane which can simultaneously attract six heavy metal *via* the double cation–π interactions.

## Conflicts of interest

There are no conflicts to declare.

## Supplementary Material

RA-009-C9RA07970J-s001
