# Supplementary material for: A novel indole-based conjugated microporous polymer for highly effective removal of heavy metals from aqueous solution via double cation–π interactions
Source: RSC Adv. 2019 Dec 6;9(69):40531–5. doi: 10.1039/c9ra07970j (PMC9076261; doi:10.1039/c9ra07970j)
Supplement: RA-009-C9RA07970J-s001 [file RA-009-C9RA07970J-s001.pdf]

Electronic Supplementary Material (ESI) for RSC Advances.
This journal is © The Royal Society of Chemistry 2019

Supporting Information

A novel indole-based conjugated microporous polymer for high effective removal of heavy metals from aqueous solution via double cation-π interactions

Qiang Wang, Rui Li, Xiao Ouyang, Guojun Wang*

Key Laboratory of Superlight Materials and Surface Technology, Ministry of Education & College of Materials Science and Chemical Engineering, Harbin Engineering University, Harbin 150001, China.

*To whom correspondence should be addressed:
Guojun Wang, Email：wang5347@hrbeu.edu.cn.

Main materials and measurements

2-indolinone was purchased from J & K Technology Co., Ltd., and used without further purification. Iron(III) chloride, Nickel (II) chloride hexahydrate ($NiCl_2 \cdot 6H_2O$), copper(II) sulfate pentahydrate ($CuSO_4 \cdot 5H_2O$), chromium(III) chloride hexahydrate ($CrCl_3 \cdot 6H_2O$) and zinc(II) sulfate hexahydrate ($ZnSO_4 \cdot 6H_2O$) were supplied by Aladdin. The rest of the materials and reagents were obtained from different commercial sources and used without further purification.

FT-IR spectrum was recorded on a Nicolet 6700 FTIR spectrometer. Solid-state cross-polarization magic-angle-spinning (CP/MAS) NMR spectra were recorded on a Bruker Avance III 400 NMR spectrometer. ^{1}H NMR and ^{13}C NMR spectra were recorded using Bruker AMX600 MHz NMR spectrometers in DMSO-d_6. The elemental analysis characterization technique was performed using a Vario EL III apparatus. UV-Visible spectroscopies were analyzed on the UV-3150 instrument. Scanning electron microscopy (SEM) was recorded on an S-4800 (Hitachi Ltd) field emission scanning electron microscope. Morphological observation was performed with a Tecnai G2 F20 S-TWIN (FEI Company) transmission electron microscope. Digital photos were taken using a Cannon 600D camera. The nitrogen adsorption and desorption isotherms were measured at 77 K using a Autosorb IQ instrument. The sample was treated at 120 °C for 24 h before the measurement. The surface area was calculated by Brunauer-Emmett-Teller

(BET) equation (0.01 < P/P0 < 0.1). The pore-size-distribution (PSD) curve was obtained from the adsorption branch using non-local density functional theory (NL-DFT) method. The total pore volume (V_{total}) and micropore volume (V_{micro}) were estimated from the adsorbed amount at a relative pressure P/P_0 of 0.995 and 0.1, respectively. The metal ion concentrations in solutions before and after adsorption were measured using ICP-AES (Jarrel-ASH, ICAP-9000) and ICP-MS (NexION 300X) for much low concentrations. For determining the compositions of the solid samples, ICP-AES (~0.1 M HNO_3 solution was used to dissolve the solids).

Heavy metal uptake experiments

The heavy metal uptakes from aqueous solutions with various concentrations were studied using the batch method. 0.02 g PTIA solid was mixed with 25 mL (V/m = 1250 mL g^{-1}) of each solution, under stirring for 24 h. After the adsorption experiments completed, centrifugation was performed, and the solid samples were dried in air for further characterization. Meanwhile, the metal concentrations in the supernatant solutions and their mother solutions were determined using inductively coupled plasma-atomic emission spectroscopy (ICP-AES) and ICP-MS for much low concentrations. The experiments were repeated three times and the result was presented as average at room temperature. The pH value of Ni(II), Cu(II), Cr(III)

and Zn(II) solutions were adjusted using 0.1 mol L^{-1} HNO_3 or 0.1 mol L^{-1} NaOH solution. Fresh prepared solutions were utilized for all experiments.

Equilibrium adsorption isotherm studies for Ni(II), Cu(II), Cr(III) and Zn(II): The concentrations of Ni(II), Cu(II), Cr(III) and Zn(II) ions were in the range of 10–500 ppm to ensure adsorption equilibration was achieved. An amount of 0.02 g PTIA added in 25 mL solution was used to have a V/m value of 1250 mL g^{-1}. The contact time was about 24 h. Adsorption kinetics of Ni(II), Cu(II), Cr(III) and Zn(II): An amount of 0.04 g PTIA was added into the 50 mL (V/m = 1250 mL g^{-1}) solution with a concentration of ≈ 10 ppm, undergoing vigorous stirring continuously for 1 min, 5 min, 30 min, 1 h, 2 h, 3 h and 4 h. The contact time was varied to check the adsorption kinetics.

Regeneration study: After the equilibrium study, metal-loaded PTIA was collected and washed with deionized water and dried to the constant weight. Then the adsorbents (0.1 g) were shaken with 50 ml of HCl (1 mol L^{-1}) in 250 mL Erlenmeyer flasks at 150 rpm on an orbital shaker at 25°C for 2 h. After washing with deionized water, the treated PTIA was ready for the next use.

Data treatment: The distribution coefficient (K_d) is defined by the equation of $K_d = (V[(C_0 - C_f)/C_f])/m$, where C_0 and C_f are, respectively, the initial and equilibrium concentrations of M^{n+} (ppm, μg mL^{-1}) after the contact, V is the solution volume (mL), and m is the solid amount (g). The % removal is calculated

with the equation of 100 × (C_0 − C_f)/C_0. The removal capacity (q_m) is given by the equation: $q_m = 10^{-3} \times (C_0 - C_f) \cdot V/m$.

Synthesis and characterization of TAT

TAT molecule was prepared with a slight modification to what was published in previous literature. [1] A mixture of 2-indolinone (2.0 g, 15 mmol) and 10 ml of $POCl_3$ was heated at 100°C for 8 h. Then, the reaction mixture was poured into ice and neutralized carefully with NaOH until pH 7-8. After neutralization, the precipitate was filtered to give the crude product as a brown solid. The crude solution in methanol (MeOH) was absorbed on silica-gel, dried, loaded and eluated through a thick silica-gel pad with a dichloromethane (DCM) as a mobile phase. After evaporation of eluate at reduced pressure and recrystallization from dimethylacetamide (DMAc), pure pale yellow solid was obtained. (0.81 g, 47 %). ^{1}H NMR (400 MHz, DMSO-*d6*) δ = 11.86 (1s, 3H, N-H), 8.65 (1d, 3H, *J=7* Hz, aromatic H),7.76 (1d, 3H, *J= 7.4* Hz, aromatic H), 7.34 (1m, 6H, aromatic H) ppm; ^{13}C NMR(100 MHz, $(CD_3)_2S{=}O$): δ = 138.98, 134.09, 122.83, 122.66, 120.29, 119.51, 111.43, 100.88 ppm; FT-IR spectrum(KBr pellet, cm^{-1}): 3436, 1608, 728. Anal. Calcd. For $C_{24}H_{15}N_3$: C, 83.46; H, 4.38; N, 12.17; Found: C, 82.58; H, 4.19; N, 12.39.

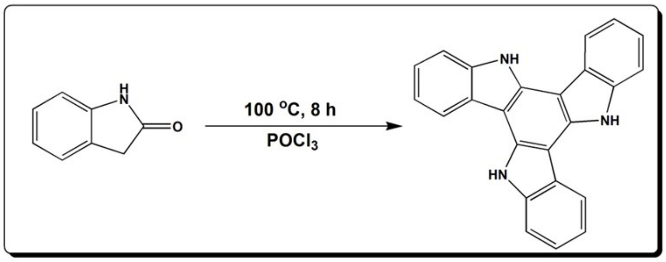

Scheme S1. Scheme for the synthesis of 10,15-dihydro- 5H-diindolo[3,2-a:3',2'-c]carbazole (TAT).

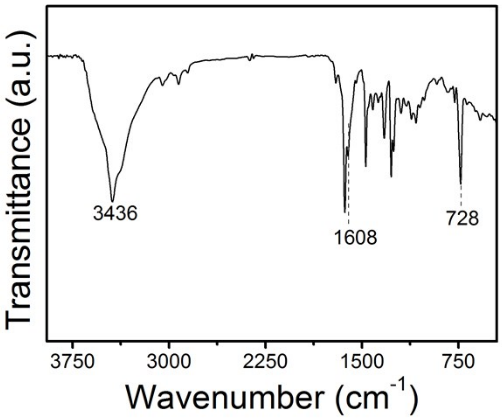

Fig. S1. FT-IR of TAT.

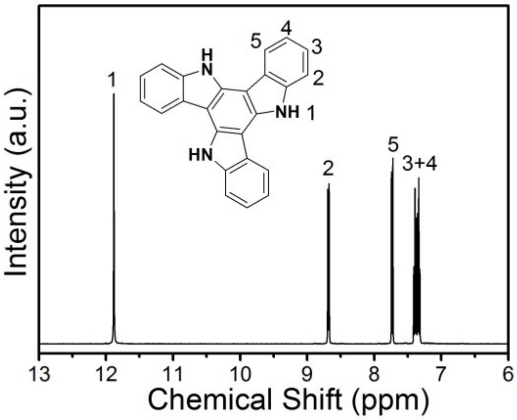

Fig. S2 ^{1}H NMR spectrum of TAT.

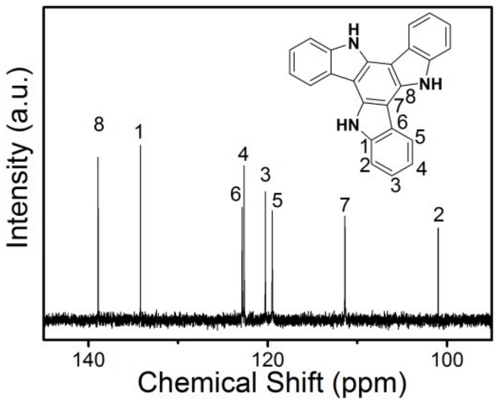

Fig. S3 ^{13}C NMR spectrum of TAT.

Preparation of the PTIA

TAT (690 mg, 2 mmol) was dispersed in 60 mL of dry $CHCl_3$ under the nitrogen environment. To this solution, we added iron(III) chloride (0.970 mg, 6 mmol), and the mixture was stirred at room temperature for 24 h. The resulting mixture was transferred to 100 mL of methanol. The precipitates were collected by filtration and washed with methanol, dilute HCl, distilled water, and methanol, sequentially. After washing, the precipitates were collected and dried under vacuum at 80 °C for 24 h and PTIA was obtained, finally.

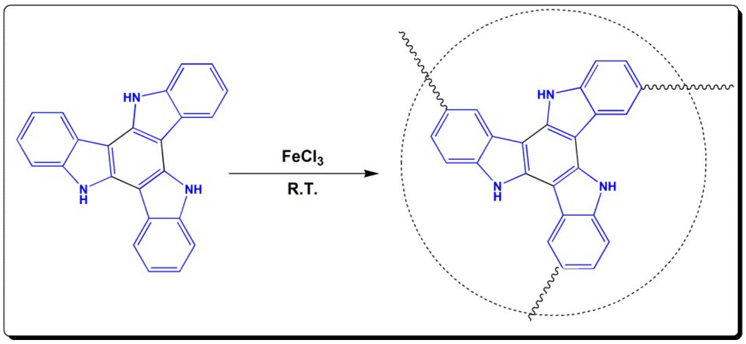

Scheme S2 Synthesis of indole-based conjugated microporous polymer **(**PTIA).

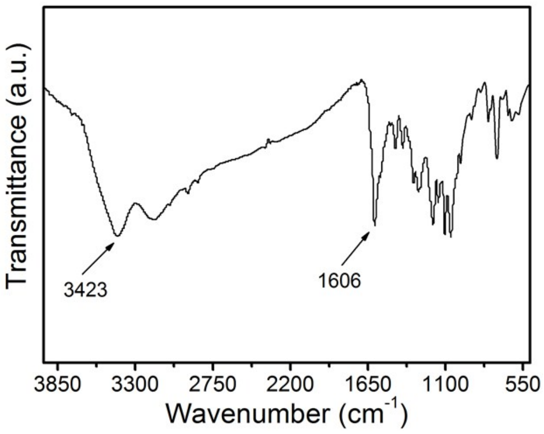

Fig. S4 FT-IR spectrum of PTIA.

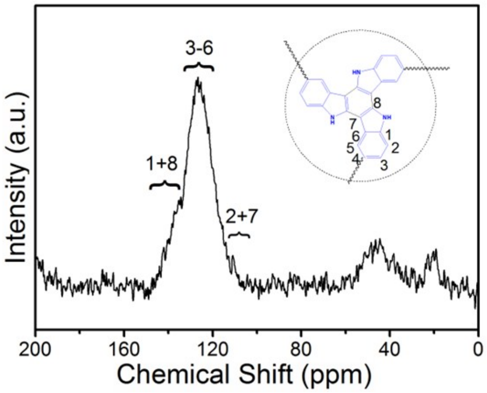

Fig. S5 ^{13}C CP/MAS NMR spectrum of PTIA.

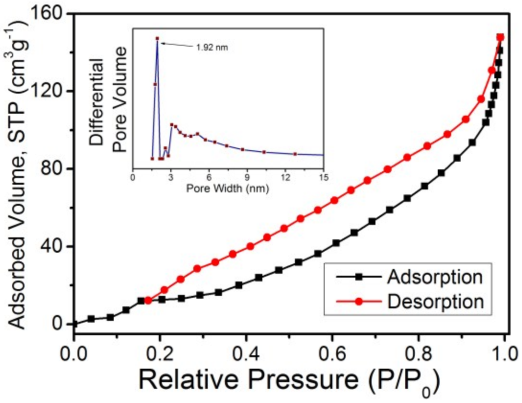

Fig. S6 N_2 adsorption-desorption isotherm curves and the pore size distribution (inset) of PTIA.

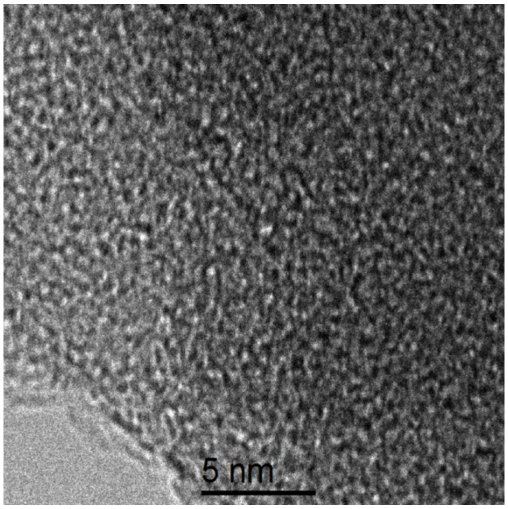

Fig. S7 TEM of PTIA network (supplement for Fig. 1b).

pH effect on the removal of Ni^{2+}, Cu^{2+}, Cr^{3+} and Zn^{2+} by PTIA

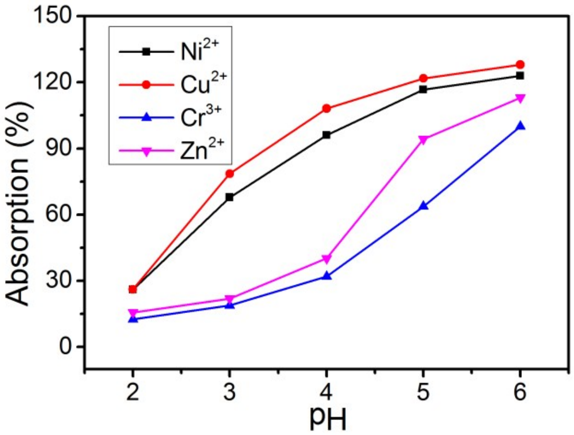

Fig. S8 pH effect on the removal of Ni^{2+}, Cu^{2+}, Cr^{3+} and Zn^{2+} by PTIA (C_0 = 100 mg/L; m = 0.02 g, V = 25 mL, V/m = 1250 mL g^{-1}; Contact time: 24 h).

Kinetics data of Ni^{2+}, Cu^{2+}, Cr^{3+} and Zn^{2+} using PTIA

Table S1. Kinetics data of Ni^{2+} using PTIA.

C_0 [ppm][a)]	Time (min)	C_f [ppm]	Removal [%]	K_d [mL g^{-1}]
9.83	1	0.272	97.23	4.4×10^4
	5	0.151	98.46	8.0×10^4
	30	0.074	99.25	1.6×10^5

	60	0.043	99.56	2.8×10^5
	120	0.031	99.68	4.0×10^5
	180	0.016	99.84	7.7×10^5
	240	0.013	99.87	9.4×10^5

[a)]m = 0.04 g, V = 50 mL, V/m = 1250 mL g^{-1}, pH value: 6.0.

Table S2. Kinetics data of Cu^{2+} using PTIA.

C_0 [ppm][a)]	Time (min)	C_f [ppm]	Removal [%]	K_d [mL g^{-1}]
10.67	1	0.17	98.41	4.2×10^4
	5	0.087	99.18	1.5×10^5
	30	0.031	99.71	4.3×10^5
	60	0.016	99.85	8.3×10^5
	120	0.013	99.88	1.0×10^6
	180	0.009	99.92	1.5×10^6
	240	0.007	99.93	1.9×10^6

[a)]m = 0.04 g, V = 50 mL, V/m = 1250 mL g^{-1}, pH value: 6.0.

Table S3. Kinetics data of Cr^{3+} using PTIA.

C_0 [ppm][a)]	Time (min)	C_f [ppm]	Removal [%]	K_d [mL g^{-1}]
9.81	1	0.335	96.59	3.5×10^4
	5	0.28	97.15	4.3×10^4
	30	0.231	97.65	5.2×10^4
	60	0.204	97.92	5.9×10^4

	120	0.185	98.11	6.5×10^4
	180	0.167	98.30	7.2×10^4
	240	0.163	98.34	7.4×10^4

[a)]m = 0.04 g, V = 50 mL, V/m = 1250 mL g^{-1}, pH value: 6.0.

Table S4. Kinetics data of Zn^{2+} using PTIA.

C_0 [ppm][a)]	Time (min)	C_f [ppm]	Removal [%]	K_d [mL g^{-1}]
10.10	1	0.289	97.14	4.2×10^4
	5	0.227	97.75	5.4×10^4
	30	0.173	98.29	7.2×10^4
	60	0.147	98.54	8.5×10^4
	120	0.125	98.76	1.0×10^5
	180	0.113	98.88	1.1×10^5
	240	0.101	99.00	1.2×10^5

[a)]m = 0.04 g, V = 50 mL, V/m = 1250 mL g^{-1}, pH value: 6.0.

Table S5. Kinetics parameters (pseudo-second-order-model) for adsorbing metal ions onto PTIA.

Ions	$q_{e,exp}$ (mg/g)	k_2	$q_{e,cal}$ (mg/g)	R^2
Ni^{2+}	12.271	0.617	12.274	1
Cu^{2+}	13.329	1.546	13.330	1

Cr^{3+}	12.059	0.547	12.061	1
Zn^{2+}	12.499	0.510	12.498	1

Sorption data of PTIA toward Ni^{2+}, Cu^{2+}, Cr^{3+} and Zn^{2+}

Table S6. Sorption data of PTIA toward Ni^{2+}.

C_0 [ppm][a)]	C_f [ppm]	Removal [%]	q_m [mg g^{-1}]	K_d [mL g^{-1}]
9.83	0.0009	99.99	12.3	1.4×10^7
51.1	0.036	99.93	63.8	1.8×10^6
99	0.56	99.43	123.1	2.2×10^5
196	2.9	98.52	241.4	8.3×10^4
356	130	63.48	282.5	2.2×10^3
518	286	44.79	290.0	1.0×10^3

[a)] m = 0.02 g, V = 25 mL, V/m = 1250 mL g^{-1}; contact time: 24 h; pH value: 6.0.

Table S7. Sorption data of PTIA toward Cu^{2+}.

C_0 [ppm][a)]	C_f [ppm]	Removal [%]	q_m [mg g^{-1}]	K_d [mL g^{-1}]
10.67	0.0005	100.00	13.3	2.7×10^7
52.7	0.026	99.95	65.8	2.5×10^6
104	0.93	99.11	128.8	1.4×10^5

209	11	94.74	247.5	2.3×10^4
345	101	70.72	305.0	3.0×10^3
508	249	50.98	323.8	1.3×10^3

a) m = 0.02 g, V = 25 mL, V/m = 1250 mL g^{-1}; contact time: 24 h; pH value: 6.0.

Table S8. Sorption data of PTIA toward Cr^{3+}.

C_0 [ppm][a)]	C_f [ppm]	Removal [%]	q_m [mg g^{-1}]	K_d [mL g^{-1}]
9.81	0.047	99.52	12.2	2.6×10^5
47.8	0.86	98.20	58.7	6.8×10^4
96	16	83.33	100.0	6.3×10^3
214	88	58.88	157.5	1.8×10^3
359	223	37.88	170.0	7.6×10^2
499	356	28.66	178.8	5.0×10^2

[a)] m = 0.02 g, V = 25 mL, V/m = 1250 mL g^{-1}; contact time: 24 h; pH value: 6.0.

Table S9. Sorption data of PTIA toward Zn^{2+}.

C_0 [ppm][a)]	C_f [ppm]	Removal [%]	q_m [mg g^{-1}]	K_d [mL g^{-1}]
10.1	0.037	99.63	12.6	3.4×10^5

53	1.5	97.17	64.4	4.3×10^4
103	12	88.35	113.8	9.5×10^3
211	68	67.77	178.8	2.6×10^3
349	193	44.70	195.0	1.0×10^3
509	346	32.02	203.8	5.9×10^2

a) m = 0.02 g, V = 25 mL, V/m = 1250 mL g^{-1}; contact time: 24 h; pH value: 6.0.

Table S10. Adsorption constants of Langmuir models for the adsorption of Ni^{2+}, Cu^{2+}, Cr^{3+} and Zn^{2+} ions onto PTIA.

Ions	q_m (mg/g)	b (L/mg)	R^2
Ni^{2+}	289.9	1.3911	0.9992
Cu^{2+}	323.6	0.5383	0.9978
Cr^{3+}	179.9	0.1421	0.9988
Zn^{2+}	205.3	0.1623	0.9999

Table S11. Comparison of adsorption capacities of various adsorbents for heavy metal ions.

target ions	adsorbents	q_m [mg g^{-1}]	References
Ni^{2+}	PTIA	289.9	This work
	PMCNa hybrid hydrogels	224	2
	Polyvinyl alcohol/corn	7.3	3

	starch hydrogel		
	Fireweed carbon	10.12	4
	Graphene – chitosan – Mn_3O_4	49.3	5
	high-pressure steaming hide waste (HWSAC)	61.92	6
Cu^{2+}	PTIA	323.6	This work
	polypyrrole/MoS_4^{2-}(MoS_4 -Ppy)	111	7
	SPI/PEI composite hydrogels	136.2	8
	EDTA-silica	79	9
	CCS-g-PGMA-c-PEI microspheres	229	10
	PEI-cellulose nanofiber	90.1	11
Cr^{3+}	PTIA	179.9	This work
	Biomass based hydrogel (SESD–PAA)	41.7	16
	nano chelating resin 2-amino pyridine−functionalized polyacrylonitrile (CPN-AP)	22	12
	PMHS-g-PyPz(OEt)2Allyl	46.8	13
Zn^{2+}	PTIA	205.3	This work
	Biomass based hydrogel (SESD–PAA)	121.2	16
	Iron Oxide (Fe_3O_4) Nanomaterial	11.1	14
	Coffee husk (CH)	12.53	15

Adsorption cycle test

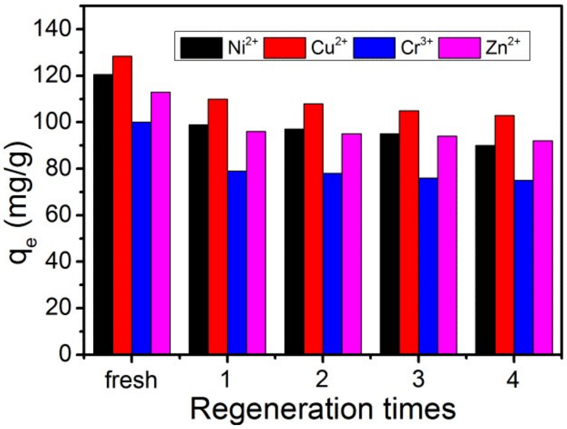

Fig. S9 Adsorption (C_0 = 100 mg/L; m = 0.02 g, V = 25 mL, V/m = 1250 mL g^{-1}; Contact time: 24 h, pH = 6) of metal ions on PTIA after repeated adsorption cycle (treated by 1 mol L^{-1} HCl).

Simulation method

The Density functional theory (DFT) calculation was utilized to investigate the adsorption mechanism. All the calculations reported here were fulfilled with Materials Studio DMol3 program (Accerlrys. USA). [17-19] For the exchange correlation term of the energy functional, the generalized gradient corrected functional GGA and PW91 functional as implemented, were applied for all the geometry optimizations.[18] The double numerical plus polarization (DNP) basis sets was employed. All the energy values were determined for 298 K. No restrictions on symmetries were imposed on the initial structures. The frequency analysis was performed on all DFT structures to ensure the absence of imaginary frequency and verify the existence of a true minimum. The computed interaction enthalpies at 298 K for the complexes were defined as,

$$-\triangle E = -\left[E_{complex} - \left(E_{Cu^{2+}} + E_A\right)\right] \quad (1),$$

where A represents PTIA.

References

[1]. L. G. Satriani, V. Casagrande, A. Bianco, G. Ortaggia, M. Franceschin, *Org. & biomol. chem.*, 2009, **7**, 2513.

[2]. P. M. Spasojevic, V. V. Panic, M. D. Jovic, J. Markovic, C. V. Roost, I. G. Popovic, S. J. Velickovic, *J. Mater. Chem. A*, 2016, **4**, 1680.

[3]. M. N. K. Chowdhury, A. F. Ismail, M. D. H. Beg, G. Hegde, R. J. Gohari, *New J. Chem.*, 2015, **39**, 5823.

[4]. A. D. Dwivedi, S. P. Dubey, M. Sillanpää, Y. N. Kwon, C. Lee, *Chem. Eng. J.*, 2015, **281**, 713.

[5]. X. Gu, Y. Yang, Y. Hu, M. Hu, C. Wang, *Acs Sustain. Chem. Eng.*, 2015, **3**, 1056.

[6]. J. Kong, R. Gu, J. Yuan, W. Liu, J. Wu, Z. Fei, Q. Yue, *Ecotox. Environ. Saf.*, 2018, **156**, 294.

[7]. L. Xie, Z. Yu, S. M. Islam, K. Shi, Y. Cheng, M. Yuan, J. Zhao, G. Sun, H. Li, S. Ma, *Adv. Funct. Mater.*, 2018, 1800502.

[8]. J. Liu, D. Su, J. Yao, Y. Huang, Z. Shao, X. Chen, *J. Mater. Chem. A*, 2017, **5**, 4163.

[9]. R. Kumar, M. A. Barakat, Y. A. Daza, H. L. Woodcock, J. N. Kuhn, *J. Colloid Interf. Sci.*, 2013, **408**, 200.

[10]. L. Lv, J. Zhang, S. Yuan, L. Huang, S. Tang, B. Liang, S. O. Pehkonen, *Rsc Adv.*, 2016, **6**, 78136.

[11]. J. Wang, X. Lu, P. F. Ng, K. I. Lee, B. Fei, J. H. Xin, J. Y. Wu, *J. Colloid Interf. Sci.,* 2015, **440**, 32.

[12]. S. M. El-Bahy, Z. M. El-Bahy, *J. Environ. Chem. Eng.*, 2016, **4**, 276.

[13]. M. Cegłowski, G. Schroeder, *Chem. Eng. J.*, 2015, **259**, 885.

[14]. S. E. Ebrahim, A. H. Sulaymon, H. S. Alhares, *Desalin. Water Treat.*, 2015, **62**, 1.

[15]. B. G. Alhogbi, Z. F. Al-Enazi, *J. Enc. Adsorpt. Sci.*, 2018, **08**, 1.

[16]. M. Zhang, L. H. Song, H. Jiang, S. Li, Y. Shao, J. Yang, J. Li, *J. Mater. Chem. A*, 2017, **5**, 3434.

[17]. W. Wang, C. Zhu and Y. Cao, *Int. J. Hydrogen Energ.,* 2010, **35**, 1951.

[18]. K. J. Lee, W. L. Mattice, R. G. Snyder, *J. Chem. Phys.*, **1992**, 96, 9138.

[19]. R. Lü, J. Lin and Z. Qu, *Comput. Theor. Chem.,* 2012, **1002**, 49.
